# Age-Related Differences in Cardiac Autonomic Control at Resting State and in Response to Mental Stress

**DOI:** 10.3390/diagnostics11122218

**Published:** 2021-11-27

**Authors:** Bernhard Grässler, Milos Dordevic, Sabine Darius, Lukas Vogelmann, Fabian Herold, Corinna Langhans, Nicole Halfpaap, Irina Böckelmann, Notger G. Müller, Anita Hökelmann

**Affiliations:** 1 Department of Sport Science, Faculty of Humanities, Otto von Guericke University, 39106 Magdeburg, Germany; corinna.langhans@ovgu.de (C.L.); nicole.halfpaap@ovgu.de (N.H.); anita.hoekelmann@ovgu.de (A.H.); 2 German Center for Neurodegenerative Diseases (DZNE), 39120 Magdeburg, Germany; milos.dordevic@uni-potsdam.de (M.D.); fabian.herold@uni-potsdam.de (F.H.); notger.mueller@uni-potsdam.de (N.G.M.); 3 Department of Neurology, Medical Faculty, Otto von Guericke University, 39120 Magdeburg, Germany; 4 Research Group Degenerative and Chronic Diseases, Movement, Faculty of Health Sciences, University of Potsdam, 14469 Potsdam, Germany; 5 Department of Occupational Medicine, Medical Faculty, Otto von Guericke University, 39120 Magdeburg, Germany; sabine.darius@med.ovgu.de (S.D.); irina.boeckelmann@med.ovgu.de (I.B.); 6 Department of Neuropsychology and Psychopharmacology, Maastricht University, 6229 ER Maastricht, The Netherlands; l.vogelmann@student.maastrichtuniversity.nl; 7 Center for Behavioral Brain Sciences (CBBS), Brenneckestraße 6, 39118 Magdeburg, Germany

**Keywords:** heart rate variability, cardiac autonomic control, aging, cardiovascular health, cognition

## Abstract

Our goal was to investigate age-related differences in cardiac autonomic control by means of heart rate variability (HRV). For this purpose, 30 healthy older and 34 younger adults were studied during three different conditions: (i) during resting state, (ii) during the execution of two cognitive tasks, and (iii) during the subsequent recovery phase. Mean heart rate and HRV parameters were higher in younger compared to older participants during all three conditions. While the mean heart rate was higher in older adults during the cognitive tasks compared to the resting state, it did not change in younger adults. In contrast, the change in HRV during the three conditions did not differ between age groups. Our results suggest decreased parasympathetic activity reflecting declined cardiac autonomic control with aging. In conclusion, HRV analysis could support the assessment of normal age-related alterations in cardiac autonomic control at resting state and in response to cognitive demands.

## 1. Introduction

Fluctuations in the time intervals between successive heartbeats are described as heart rate variability (HRV) [1]. These fluctuations are the result of several and complex physiological processes. Short-term HRV (~5 min) is generated by two sources [2]. The first one includes the complex interaction between the two branches of the autonomic nervous system (ANS), i.e., sympathetic and parasympathetic branch. Sympathetic activity increases heart rate and parasympathetic activity decreases heart rate [3]. In contrast to the earlier concept that an increase in sympathetic activity automatically leads to a decrease in parasympathetic activity and vice versa [4], it is now known that both the sympathetic and the parasympathetic systems can be active simultaneously. Moreover, an increase in the activity in one system does not necessarily lead to a decreased activity in the other system [5]. Sudden changes in heart rate are primarily parasympathetic mediated and therefore, short term HRV is mainly determined by parasympathetic influences [3]. Regulatory mechanisms controlling heart rate are the second source, i.e., respiratory sinus arrhythmia (RSA), baroreceptor reflex, and rhythmic changes in vascular tone [2]. RSA describes the phenomenon that heart rate increases during inspiration and decreases during expiration [6]. A pronounced RSA with slow, deep breathing characterizes a healthy organism [5]. Baroreceptors measure the blood pressure in the aortic branch and arteries, and induce changes in heart rate and vascular tone through feedback mechanisms [7]. HRV finally reflects all these dynamic and nonlinear physiological processes and can therefore be considered as a measure of neurocardiac function reflecting heart–brain interactions and ANS dynamics [6].

HRV can be quantified using time-domain, frequency-domain, and non-linear parameters [1,2,8]. A very simple method to describe the overall HRV is the calculation of the standard deviation of the NN intervals (SDNN). It describes all cyclic components contributing to variability [1]. Indices primarily reflecting vagally-mediated (parasympathetic) changes in HRV are RMSSD (root mean square of successive NN interval differences) and HF (power of the high-frequency band, 0.15–0.4 Hz) [9]. Higher values indicate healthy functioning of cardiac autonomic control, high self-regulatory capacity, adaptability, and resilience [3]. Contrarily, relatively low values indicate chronic stress, inadequate functioning of self-regulation, and higher risk for cardiovascular diseases [3,10]. In contrast to HF, the interpretation of the parameter LF (power of the low-frequency band, 0.04–0.15 Hz), indicating long-term HRV, is less clear. It is assumed that it is not a mere index of sympathetic regulation of heart rate but reflects baroreceptor activity [1,2,3]. Since physiological systems rarely work in a linear way, heart rate complexity can be described by means of non-linear parameters [11], which has rarely been used in psychophysiological research. Among them, correlation dimension (D2 or CD) is a measure of the self-similarity of NN intervals [11]. Lower values indicate a shift towards sympathetic regulation of the ANS. In contrast, higher values reflect complex behavior of heart rate regulation, which can be interpreted as greater adaptability and a healthy ANS [11].

Age-related degradation processes increase the risk of cardiovascular diseases [12]. Among them are a decrease in parasympathetic and an increase in sympathetic activity [13]. Loss of vagal activity, characterized as autonomic imbalance, is associated with increased morbidity and higher risk of cardiovascular diseases [10,14,15,16,17].

The connection between brain and heart also implies a relationship between cardiac autonomic control and cognitive processes. Several models postulate this neurovisceral relationship and focus on vagal modulation as their common factor [9]. The most popular model is the neurovisceral integration model of Thayer et al. [18]. The neurovisceral integration model postulates that cardiac autonomic control and higher cognitive performance relies, at least partly, on the same neural correlates (e.g., prefrontal cortex). Based on this model, the prefrontal cortex is, among other cerebral structures (e.g., anterior cingulate cortex, insular cortex, amygdala) part of the so-called central autonomic network (CAN) being involved in cognitive processes and heart rate regulation [18]. Moreover, it has been shown that prefrontal activity is associated with vagally-mediated HRV and cognitive performance is related to HRV at resting state (for review see [18]). 

A positive relationship between cognitive performance and HRV has been evidenced in the literature [19,20]. In this context, it has been recommended that a thorough evaluation of the relationship between cardiac autonomic functioning and cognitive performance includes the assessment of resting, reactivity, and recovery HRV [9,21]. Within this framework, reactivity refers to the change between resting state and event (e.g., cognitive tasks) [22]. However, studies on HRV reactivity and recovery in psychophysiological research are currently relatively scarce and mainly focus on young adults [23,24,25,26]. Age-related changes in resting HRV have been investigated in several studies and a decrease in HRV, mainly in vagally mediated HRV parameters, has been frequently reported [13,27,28,29,30,31,32,33]. However, there is a gap in the literature regarding the investigation of age-related changes in HRV in response to cognitive tasks. Only a few studies have been conducted investigating age-related effects on cardiac autonomic control (i.e., operationalized by HRV) at resting state, during the cognitive tasks, and in a recovery period after the cognitive testing [34,35]. Moreover, the available studies only considered changes in mean heart rate and frequency-domain parameters but did not assess time-domain and non-linear parameters.

This study aims to investigate the effects of age group and condition (resting state, during the cognitive tasks, and in a recovery phase after the cognitive testing) on cardiac autonomic control. For this purpose, the HRV of young (YA) and older adults (OA) was measured by means of electrocardiography (ECG) before, during, and after two different cognitive tasks: Stroop and N-back. Before and after the task, standardized, 5-min resting state measurements were performed. The Stroop task is a widely used task assessing the executive functions inhibitory and cognitive control [36,37]. Working memory capacity was assessed using the N-back task [38].

As stated above, HRV declines with increasing age. Therefore, we expected lower resting HRV in OA compared to YA. Based on previous research, we further expected lower HRV in the recovery phase due to a slower recovery of parasympathetic activity and a more pronounced sympathetic activity during the cognitive tasks in OA compared to YA [35]. Interpretation of HRV during cognitive tasks and HRV reactivity is more challenging compared to the interpretation of HRV obtained at resting state as it depends on the task difficulty [9]. A sharp decrease in HRV during tasks that do not require executive functions indicates a high ability to adapt to the situation [9]. In contrast, vagal withdrawal should be low during tasks that require high executive demands [39]. Since we assume lower cognitive capacities in OA compared to YA [40,41], and thus a higher mental workload for OA compared to YA, we expected different HRV responses with respect to the cognitive load. Based on the assumptions of Laborde et al. [9] and Thayer et al. [39], we hypothesized a pronounced decrease of HRV during conditions requiring low executive demands (Stroop congruent and 0-back) in YA compared to OA. In contrast, in conditions requiring high executive demands (Stroop incongruent, mixed, and 2-back), we expected no (further) vagal withdrawal in YA compared to OA.

## 2. Materials and Methods

### 2.1. Participants

**Figure 1:**
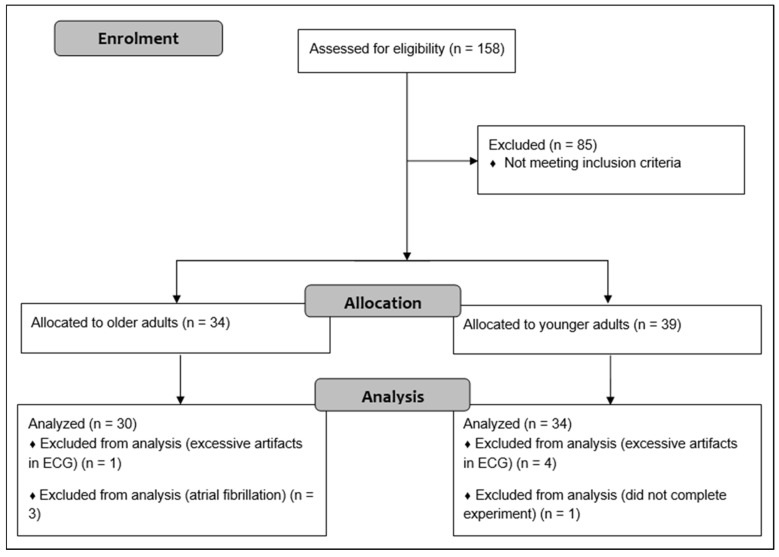
Flow diagram describing flow of participants.

A total of 158 adults were initially recruited through advertisements in local newspaper, flyers, posters, and using existing databases. The required sample size was determined on the basis of prior research comparing the resting state HRV of older with younger adults [35]. According to the means and standard deviations of ln HF, a sample size of 16 participants per group was calculated to achieve a power of 0.95. G*Power 3.1 software was used for these calculations [42]. Given that this study included 33 younger and 32 older adults, we intended to include at least 30 participants per group. OA were administered the CERAD plus test battery to detect the presence of cognitive impairment [43]. Participants who scored 1.5 z-scores below the age- and education-adjusted reference sample in at least one subtest of the CEARD plus test battery were not included in the healthy, older group but were referred to experienced neurologists for further diagnosis and eventually participated in further studies. The inclusion criteria were as follows: 18 to 30 or 54 to 80 years of age; native German-speaking; and living and able to manage everyday activities independently. Exclusion criteria were: neurological diseases (e.g., epilepsy, multiple sclerosis); severe cardiac diseases (e.g., severe cardiac insufficiency, cardiac pacemaker, valvular defect, arterial hypertension, cardiac arrhythmias, atrial fibrillation); mental diseases (e.g., schizophrenia, depression (score > 5 in the Geriatric Depression Scale (GDS)); orthopedic diseases (e.g., bone fracture in last six months, symptomatic slipped disc); muscular diseases (e.g., myositis, tendovaginitis); severe endocrinologic diseases (e.g., manifest hypothyroidism or hyperthyroidism, adiposity (BMI > 30), insulin dependent diabetes mellitus type II); injury or surgery in the last six months; consumption of illegal intoxicants or alcohol abuse; uncorrected poor eyesight or hearing; anamnestic known color blindness or red-green weakness; pregnancy or breastfeeding; and using beta blockers, neuroleptics, narcotic analgesics, benzodiazepines, or psychoactive medications. After exclusion of participants that did not meet our criteria, 39 YA and 34 OA were included in the study. Finally, five YA and two OA were excluded from the analysis due to a high number of artifacts in the ECG (>5% in at least one condition), atrial fibrillation (*n* = 3), and one participant did not complete the whole experiment. The older and younger groups were matched for gender. Flow diagram of the study is shown in Figure 1.

### 2.2. Experimental Design

All measurements were conducted in laboratories at the Otto von Guericke University Magdeburg and prior to the experiment, all participants were briefed about the experimental procedure. After being informed of possible risks and benefits associated with the study, participants provided written consent to participate. 

Prior the HRV experiment, participants were requested to refrain from intense physical training and drinking alcohol 24 h before the measurement. Drinking caffeinated drinks, smoking, and eating were not allowed two hours before the experiment to limit potential acute effects on HRV. ECG data were collected at resting state, while participants performed the cognitive tasks, and during a recovery phase after the cognitive testing. The resting state and recovery measurements lasted for five minutes each. The cognitive tasks lasted for about 30 min. A more detailed description of the study procedures can be found elsewhere [44].

### 2.3. Cognitive Tasks

Modified versions of two executive functions tasks were administered in a fixed order via a computer using the software Presentation (Neurobehavioral Systems Inc., San Francisco, CA, USA). The Stroop task consisted of three conditions with three blocks in each condition, separated by resting blocks. Each task and resting block lasted for 60 s. Four different color-words were presented and each color had a corresponding button: “RED”, “GREEN”, “BLUE”, or “YELLOW” in German. In each block, 20 color-words were consecutively presented in the middle of the screen. The first, and easiest condition, was the congruent condition. The meaning of the word and ink color matched. In the incongruent condition, meaning and ink color differed, e.g., the word “RED” was presented in blue color. Participants had to identify the color of the word and ignoring the meaning by pressing the blue button. The maximum time window in which the participants could respond was 2.5 s. Finally, in the mixed condition, 50% congruent and 50% incongruent stimuli appeared, and participants were instructed to react on the color of the word. 

The second task, N-back, also consisted of three conditions with three blocks in each condition and 20 stimuli in each block. Each task and resting block lasted for 40 s. Single-digit numbers appeared consecutively on the screen. In the 0-back condition, participants had to press the target button when the number “7” appeared. In the 1-back condition, participants had to press the target button when two identical numbers appeared in a row. In the 2-back condition, the target was any number identical to the second-last number presented before. In all three conditions, 25% of the stimuli were targets.

Participants were advised in both tasks to react as quickly and as correctly as possible. Task performance was evaluated by the percentage of errors and mean reaction time of correct responses. Wrong responses, no responses, or responses below a reaction time of 100 ms were considered as errors. Prior to the experiment, participants were allowed to practice both tasks to adequately familiarize them with the procedures. A detailed description of the procedure of the cognitive tasks can be found elsewhere [44]. 

### 2.4. Autonomic Data Collection and Processing

Electrocardiographic data were recorded using a three-channel Holter-ECG with a sampling rate of 1000 Hz (Medilog AR12plus, Schiller Medizintechnik GmbH, Baar, Switzerland). The raw data were transferred to the Medilog Darwin 2 analysis software package (Schiller Medizintechnik GmbH, Baar, Switzerland), checked automatically and visually by a healthcare professional for clinical abnormalities, and converted into text files comprising consecutive NN intervals. HRV analysis was performed using Kubios premium 3.3 software package (University of Kuopio, Kuopio, Finland). Artifact correction was done in Kubios according to national [8] and international guidelines [1] with an artifact identification threshold of 0.3 s and a smoothness priors method for detrending NN intervals (Lambda = 500, f_c_ = 0.035 Hz).

HRV was analyzed by time and frequency and non-linear methods. SDNN and RMSSD (ms) were used as time-domain, LF and HF (ms^2^) as frequency-domain, and correlation dimension D2 as non-linear measures. The frequency-domain parameters were extracted through autoregressive modeling using model order 16, as recommended in Laborde et al. [9]. According to the recommendations of Heathers [45], HF is used for measurements with a duration of at least 60 sec and LF for measurements with a duration of at least 5 min. Therefore, LF was calculated only for the resting state and recovery measurements. An advantage of D2 is that it does not need a large time series [46]. Therefore, we considered D2 for the evaluation of cardiac autonomic regulation. For on-task HRV analysis, the three blocks in each condition were averaged. In addition to HRV parameters, mean heart rate (mHR) was also reported for each condition.

Resting state and recovery measurements were conducted according to recent recommendations [9]. Participants were sitting on a comfortable chair in front of a computer screen, knees were bent at 90° angle, hands on their thighs, eyes closed, advised to relax and breath normally. To avoid artifacts, participants were advised not to move or talk during the measurement. Resting state and recovery measurement lasted for five minutes with a stabilization period prior measurement to ensure a relaxed state. 

### 2.5. Statistical Analysis

All datasets were checked for normality by Shapiro–Wilk’s test. Independent samples *t*-test were used to compare demographic and behavioral data between age groups. The sex distribution between the groups was analyzed using the Chi-squared test. Effect size d was calculated and indicated as small (d > 0.2), medium (d > 0.5), and large (d > 0.8) [47]. To test the age group × condition interaction effects, we performed 2 × 5 repeated measures analysis of variance (ANOVA) with group (YA and OA) as between-participants factor and condition (resting state, task condition, and recovery) as within-participants factor, with pair-wise comparison using Bonferroni adjustment for multiple comparisons. The Mauchly Test was used to check sphericity and, when appropriate, utilizing Greenhouse–Geisser corrected *p* values. For significant results, we reported η_p_^2^ and indicated as small (η_p_^2^ > 0.01), medium (η_p_^2^ > 0.06), and large (η_p_^2^ > 0.14) effects. SPSS version 26.0 statistics package (SPSS Inc., Chicago, IL, USA) was used for statistical analysis. *p* < 0.05 was considered statistically significant, two-sided.

### 2.6. Ethical Approval

This study was approved by the Ethics Committee of the Otto von Guericke University Magdeburg (reference number: 83/19) and is in accordance with the latest version of the Declaration of Helsinki. The study was registered in ClinicalTrials.gov (NCT04427436) on the 10 June 2020.

## 3. Results

### 3.1. Demographic and Behavioral Data

Table 1 shows the general characteristics and behavioral data of both groups. OA had a higher age (*p* < 0.001, d = 3.650) and body mass index (*p* = 0.014, d = 0.357), and a lower height (*p* < 0.001, d = 1.995) than YA. OA reacted significantly slower than YA in Stroop congruent (*p* < 0.001, d = 2.530), Stroop incongruent (*p* < 0.001, d = 2.639), Stroop mix (*p* < 0.001, d = 2.271), 0-back (*p* < 0.001, d = 1.163), 1-back (*p* < 0.001, d = 1.054), and 2-back (*p* = 0.003, d = 0.779). YA had significantly more errors in 0-back (*p* = 0.023, d = 0.548). In contrast, OA had a significantly higher error rate in the 2-back task (*p* = 0.002, d = 1.021). 

### 3.2. Autonomic Data

**Figure 2:**
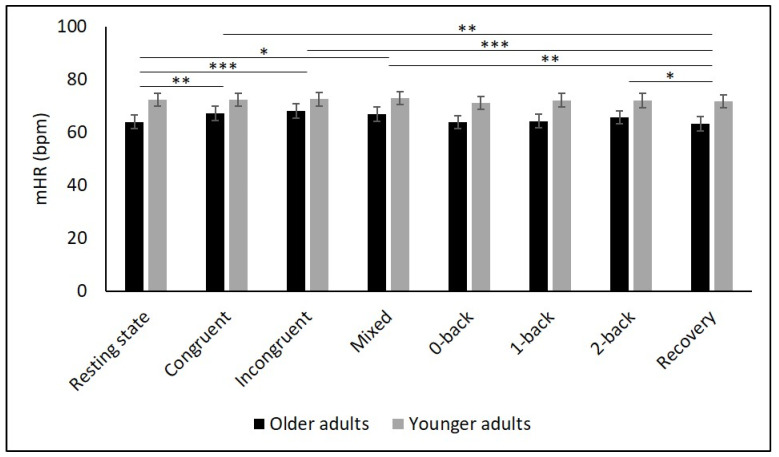
Means and group × condition interactions for mHR. Error bars indicate ±2 standard error of mean; abbreviations: bpm: beats per minute, mHR: mean heart rate. * *p* < 0.05; ** *p* < 0.01; *** *p* < 0.001.

**Figure 3:**
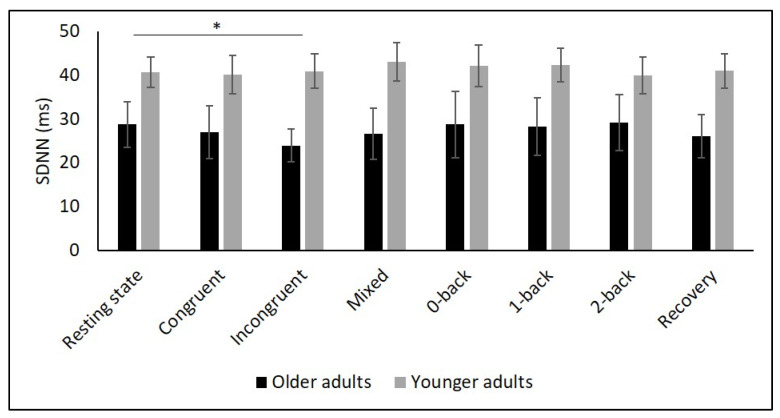
Means and group × condition interactions for SDNN. Error bars indicate ±2 standard error of mean; abbreviations: ms: milliseconds; SDNN: standard deviation of the NN intervals. * *p* < 0.05.

**Figure 4:**
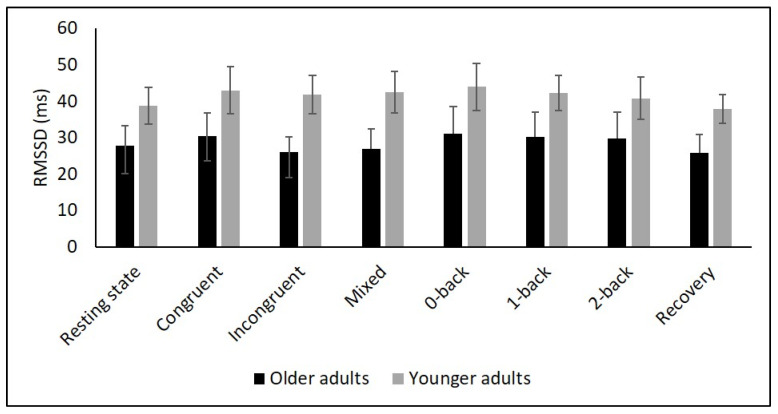
Means for RMSSD. Error bars indicate ±2 standard error of mean; abbreviations: ms: milliseconds; RMSSD: root mean square of successive NN interval differences.

**Figure 5:**
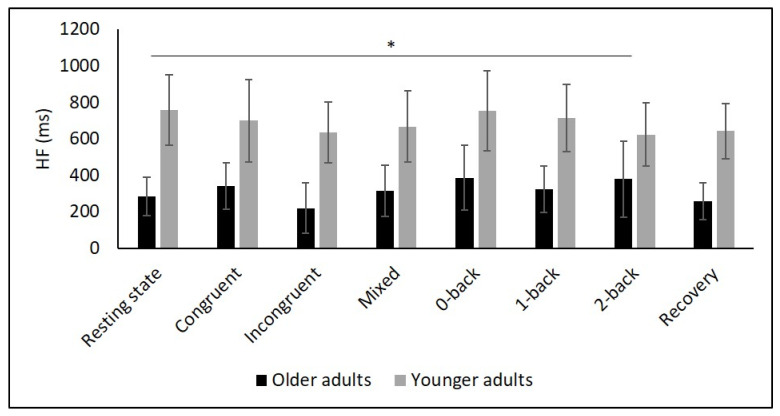
Means and group × condition interactions for HF. Error bars indicate ±2 standard error of mean; abbreviations: HF: high frequency; ms: milliseconds. * *p* < 0.05.

**Figure 6:**
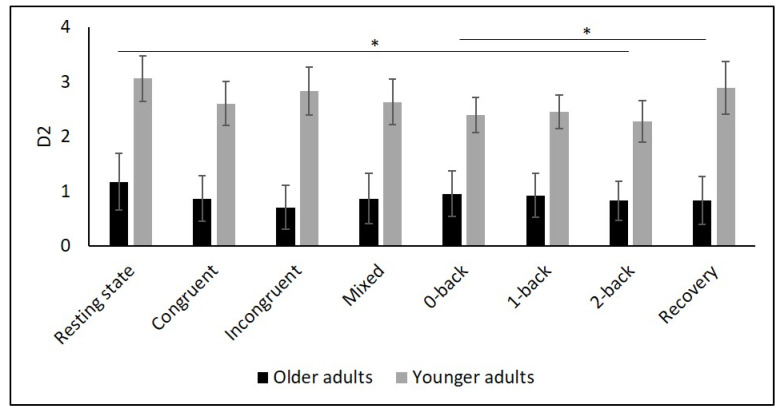
Means and group × condition interactions for D2. Error bars indicate ±2 standard error of mean. * *p* < 0.05.

Table 2 and Figure 2, Figure 3, Figure 4, Figure 5 and Figure 6 show the results of mHR and the HRV for both groups. mHR and HRV parameters were higher in YA compared to OA. Results of ANOVA indicated significant age group × condition interaction effect on mHR for Stroop (*p* < 0.001, η_p_^2^ = 0.126). Post hoc analysis for mHR revealed significant differences between resting state and Stroop congruent (*p* = 0.004, η_p_^2^ = 0.126), Stroop incongruent (*p* < 0.001, η_p_^2^ = 0.215), and Stroop mix (*p* = 0.024, η_p_^2^ = 0.080). Post hoc analysis also revealed significant differences for mHR between recovery and Stroop congruent (*p* = 0.004, η_p_^2^ = 0.127), Stroop incongruent (*p* < 0.001, η_p_^2^ = 0.237), and Stroop mix (*p* = 0.005, η_p_^2^ = 0.119). Significant results were also detected between resting state and Stroop incongruent for SDNN (*p* = 0.032, η_p_^2^ = 0.072), resting state and 2-back for HF (*p* = 0.030, η_p_^2^ = 0.074), recovery and 0-back (*p* = 0.040, η_p_^2^ = 0.066), as well as 2-back (*p* = 0.042, η_p_^2^ = 0.065) for D2 (Table 3).

## 4. Discussion

The purpose of this study was to determine the effects of age on cardiac autonomic control as well as the interaction effects between age and different conditions, namely (i) resting state, (ii) cognitive tasks, and (iii) recovery period after cognitive tasks. Global HRV (i.e., SDNN), parasympathetic mediated (i.e., RMSSD), short-term (i.e., HF), and non-linear HRV measures (i.e., D2) as well as mHR were higher in YA compared to OA during all three condition. While mHR remained relatively constant across all conditions in YA, it significantly increased in OA from resting state to all Stroop conditions, followed by a decrease in the recovery phase. In contrast, there were no interaction effects for any of the HRV parameters.

### 4.1. Effect of Age on Behavioral Data

The present study revealed slower reaction times in OA compared to YA in all six conditions supporting previous studies [35,48,49,50,51,52,53]. Error rate was significantly higher in OA only in the 2-back task. YA even produced more errors in the 0-back task. Previous studies found significantly higher error rates in OA in tasks with high cognitive demands [35,48], but also no differences in tasks with low cognitive demand, such 0- and 1-back [35,50]. Although both groups were asked to complete the tasks as accurately and as quickly as possible, it might be that YA focused more on a rapid response than on error-free completion of the task whereas OA compensated for their cognitive deficits by slower reaction times. In addition, older adults show enhanced activation of prefrontal areas [49] and enhanced cardiovascular costs [35] compared to YA. Impairment of cognitive functioning in older age might be caused by age-related loss of neurons in cortical structures (e.g., dorsolateral prefrontal cortex) [54].

### 4.2. Effects of Age on Mean Heart Rate

There is mixed evidence in the literature concerning age-related changes of mHR. While one review [55] presented studies that showed either an increase or a decrease in mHR with age, other studies reported no differences in mHR between YA and OA [29,31,34,35,56]. These divergent results could be the result of the decrease of the parasympathetic influence on the sinus node leading to an increase in heart rate [57], and, on the other hand, of a decrease in intrinsic heart rate with aging [58]. Hypothetically, both mechanisms could cancel each other out, thereby leaving the heart rate unchanged with aging.

The present study showed higher mHR in YA compared to OA during all conditions, contradicting previous studies reporting higher mHR in OA during cognitive tasks [34,35,56] or during a recovery period [35]. Compared to YA, mHR in OA increased from resting state to the cognitive tasks and decreased to the recovery period. This interaction effect in mHR was also found in a previous study using the 2-back task [35], but was not detected in other studies [34,56]. An increase in mHR in response to a cognitive load might be related to the reallocation of resources in the central nervous system in order to maintain specific information in memory [59]. Furthermore, OA attempted to compensate for their decline in cognitive performance by increasing their effort which is mirrored in an increase in sympathetic activation leading, in turn, to a higher mHR [35]. The decrease in mHR in the recovery period could be the result of a parasympathetic recurrence, indicative of healthy cardiac adaptability [35]. However, due to the significant but rather small changes in absolute values in mHR in OA, the results must be interpreted cautiously, since it must be questioned whether they are biologically relevant. The absence of changes in mHR in YA between conditions contradicts the results of other studies [35,56]. Presumably, the cognitive demand was not high enough to provoke enough stress to induce an increase in mHR in YA. Therefore, other, more difficult cognitive tasks should be considered when evaluating autonomic functioning in YA.

### 4.3. Effects of Age on Cardiac Autonomic Control

Age-related impairment in autonomic functioning is mainly mediated by a decline of the parasympathetic control over heart rate [57,60,61] and probably reflects the loss of neurons in the brain and spinal cord [3]. As a consequence, signal transmission between brain and heart is impaired and the regulatory capacity is reduced [3] which, in turn, can increase the risk of cardiovascular morbidity and mortality [14], gastrointestinal disorders, inflammation, and hypertension [62,63]. The role of changes in sympathetic activity during aging is not fully clear, as there is evidence for a decrease [60,64] as well as an increase of sympathetic activity [65] during aging. However, by means of HRV analysis, parasympathetic activity can be better assessed because LF (power in the low-frequency range; 0.04–0.15 Hz) represents a mixture of sympathetic and parasympathetic activity [1,66]. Therefore, the parameter LF should be interpreted cautiously. Instead, RMSSD and HF are parameters primarily reflecting parasympathetic activity [9]. We also assessed the non-linear parameter D2, which provides a more differentiated picture of heart rate complexity than the traditional linear HRV parameters [11].

Several studies confirmed this autonomic dysfunction in OA by showing reduced vagally mediated HRV in OA compared to YA at resting state [29,30,34,35,67,68]. As hypothesized, we observed lower SDNN, indicating lower overall HRV, and lower RMSSD as well as HF values in OA compared to YA during the resting state measurement indicating lower short-term HRV. This finding is consistent with the idea that cardiac autonomic regulation declines with age [34] being reflected in a decrease in vagally mediated HRV parameters. The higher values in LF in YA might indicate higher sympathetic, but also higher long-term HRV in YA compared to OA, and are consistent with previous studies [29,35,68]. Furthermore, D2 was lower in OA, which buttress the idea of more severe autonomic dysfunction in OA.

To comprehensively assess changes in autonomic control, it is, as recommended by Laborde et al. [9], necessary to quantify resting, reactivity, and recovery HRV. To date, only a few studies have considered this approach in YA and OA. In particular, respiratory sinus arrhythmia (RSA), a marker of vagal control, was assessed in two studies using a Stroop [56] and a working memory task [34]. In both studies, RSA was higher in YA during the tasks compared to OA. Another study reported higher HF values in YA during a 0- and 2-back task [35]. In line with these findings, we observed higher values of SDNN, RMSSD, HF as well as D2 in YA compared to OA during Stroop and N-back tasks. In contrast, our interaction analysis did not reveal age-related differences in overall or short-term HRV. This result is in line with the findings of Capuana et al. [34] but contradicts the observations of Schapkin et al. [35] as the latter study reported an interaction effect for HF.

Whether a task-induced decrease in short-term HRV is linked to a successful task completion depends on the nature of the cognitive task. For non-executive tasks, a strong decrease in HRV is associated with better task performance, whereas for difficult tasks, a small decrease in HRV is linked to a superior task performance [9]. However, this interpretation is not universal and has been critically discussed in the literature [69]. There are hints that during more difficult tasks, relatively low HRV represents efficient adaptation [69]. Interestingly, overall (i.e., SDNN) and short-term (i.e., HF) HRV in the present study did not decline from low to high cognitive load contradicting other studies reporting significant differences between tasks with low and high cognitive load [25,35]. Given that the decline in HRV in this study was only small, we speculate whether the cognitive demand was high enough to induce a significant withdrawal of parasympathetic activity.

Concerning cardiac recovery, previous studies noticed a faster recovery in YA compared to OA after cognitive tasks [69,70,71]. In the present study, YA showed higher overall, short-, and long-term HRV during recovery compared to OA, but there was no difference regarding the autonomic response from task to recovery between the age groups. This result is consistent with the results of Capuana et al. [34]. However, another study reported significant effects in such a way that OA showed a sharp decrease in HF in response to a high cognitive load (2-back task) and a smaller increase in the recovery period compared to YA [35]. One reason for the absence of interaction in the present study might be the different time periods of task conditions and resting measurements as the task conditions were shorter than the resting measurements. Assessment of sympathetic activity can be performed by measuring LF or blood pressure. However, since LF reflects both sympathetic and parasympathetic activity, this parameter is controversial [66]. Measurement of blood pressure was used in the study of Schapkin et al. [35]. Sympathetic activation (increase in systolic blood pressure) combined with parasympathetic decline (decrease in short-term HRV) is interpreted as a “reciprocal activation mode” and is considered as mobilization of resources for coping with stress [72]. Thus, simultaneous measurement of heart rate and blood pressure allows for a more comprehensive assessment of baroreflex sensitivity providing further information about blood perfusion to organs including the brain [73].

### 4.4. Strengths and Limitations

This study comprehensively assessed cardiac autonomic control by means of linear- and non-linear HRV indices in YA and OA during resting state, cognitive tasks, as well as a recovery period. Linear parameters included indices measuring overall HRV (i.e., SDNN), short- (i.e., HF), and long-term HRV (i.e., LF), as well as indices reflecting parasympathetic modulation of cardiac control (i.e., RMSSD). Moreover, both cognitive tasks comprised three conditions with different levels of difficulty allowing the assessment of cardiac autonomic control during different states of mental stress. Finally, assessment of the non-linear HRV parameter D2 may provide novel information on cardiac autonomic control compared to the traditional linear HRV parameters.

Several limitations of the study should be mentioned. Firstly, the study is limited by its small sample size. A larger sample may elicit significant interaction effects. Additionally, although there was no difference in the gender distribution between both groups, more females were recruited in both groups. This could have potentially biased the HRV responses. Future studies should consider possible differences in HRV responses between female and male participants when designing the sample. Secondly, the duration of resting measurements and task conditions differed, which could have biased the results. Due to the length of cognitive tasks (i.e., below five minutes), we refrained from analyzing LF during the tasks. However, considering LF would provide the assessment of long-term HRV. Thirdly, the recovery measurement was only conducted once after all cognitive tasks. Recovery measurements after each cognitive task would provide more detailed information about recovery ability. However, this procedure would also increase the total time spent on the investigation. Furthermore, the measurements were only done in healthy participants. Therefore, the results are not applicable to cardiac patients. The study setting for the participants was quite strict. However, this high degree of standardization is necessary to compare study results and to acquire evidence before clinical use. In addition, longitudinal studies could reveal the causal relationship between age-related changes in cognitive performance and cardiac autonomic control. Finally, measurement of blood pressure would allow the assessment of baroreflex sensitivity providing a more comprehensive picture of autonomic adaptation.

## 5. Conclusions

The main result of the present study was that healthy OA compared to YA showed impaired cardiac autonomic control. This was evident in reduced overall (i.e., SDNN), short- (i.e., HF), and long-term (i.e., LF), parasympathetic-mediated (i.e., RMSSD), as well as non-linear HRV (i.e., D2) at resting state, during cognitive tasks, and in a recovery period. These findings provide support for the idea that autonomic functioning declines with aging. However, the two age groups did not differ in terms of HRV reactivity. Thus, cardiac autonomic adaptability seems to be well maintained in the present sample of healthy adults. In contrast, mHR was higher in younger compared to older participants and there was an interaction effect showing stronger reactions of mHR in older participants in response to cognitive tasks. These results suggest greater sympathetic activity in response to the cognitive load in OA compared to YA. However, due to the small absolute changes in mHR in OA, the results should be interpreted cautiously.

Further studies should be conducted to investigate the influence of cardiac and autonomic diseases on cardiac autonomic control. As this study involved only participants with healthy cardiovascular function, the present findings are applicable only to this population. As the results of heart rate and HRV in the present study differed, further studies should investigate the sensitivity of both measures in response to mental stress in OA and YA. The present results are promising as the non-invasive measurement of cardiac autonomic control by means of HRV could support the assessment of normal age-related alterations in cardiac autonomic control at resting state and during tasks inducing mental stress.

## Figures and Tables

**Table 1:** Demographic and cognitive data (Mean ± SD).

Variable	OA (*n* = 30)	YA (*n* = 34)	*p*-Value	Effect Size d
Age (years)	68.3 ± 6.6	24.2 ± 2.9	<0.001 ^a^***	3.650
Male/female (n)	6/24	11/23	0.264 ^b^	0.140
Height (cm)	164.67 ± 6.37	173.62 ± 10.45	<0.001 ^a^***	1.995
Bodyweight (kg)	67.48 ± 11.22	68.75 ± 11.05	0.650 ^a^	0.020
BMI (kg/m^2^)	24.82 ± 3.42	22.78 ± 3.02	0.014 ^a^*	0.357
Reaction time Stroop congruent (ms)	912 ± 170	590 ± 73	<0.001 ^a^***	2.530
Error rate Stroop congruent (%)	1.50 ± 1.34	1.67 ± 1.59	0.594 ^a^	0.115
Reaction time Stroop incongruent (ms)	1145 ± 275	623 ± 84	<0.001 ^a^***	2.639
Error rate Stroop incongruent (%)	3.11 ± 3.93	2.40 ± 1.60	0.359 ^a^	0.242
Reaction time Stroop mix (ms)	1034 ± 265	603 ± 77	<0.001 ^a^***	2.271
Error rate Stroop mix (%)	1.67 ± 2.14	1.83 ± 1.58	0.729 ^a^	0.086
Reaction time 0-back (ms)	462 ± 59	397 ± 53	<0.001 ^a^***	1.163
Error rate 0-back (%)	0.00 ± 0.00	0.20 ± 0.50	0.023 ^a^*	0.548
Reaction time 1-back (ms)	478 ± 93	401 ± 70	<0.001 ^a^***	0.944
Error rate 1-back (%)	0.17 ± 0.67	0.08 ± 0.32	0.491 ^a^	0.175
Reaction time 2-back (ms)	564 ± 116	483 ± 92	0.003 ^a^**	0.779
Error rate 2-back (%)	3.39 ± 3.85	0.63 ± 0.84	0.001 ^a^**	1.021

BMI: body mass index, OA: older adults, YA: young adults. Continuous variables expressed as mean ± standard deviation, categorial variables as n. ^a^ Student’s *t*-test; ^b^ Chi-squared test. * *p* < 0.05; ** *p* < 0.01; *** *p* < 0.001.

**Table 2:** Descriptive results for mHR and HRV measures (Mean ± SEM).

	mHR (bpm)		SDNN (ms)		RMSSD (ms)		HF (ms^2^)		LF (ms^2^)		D2	
	OA	YA	OA	YA	OA	YA	OA	YA	OA	YA	OA	YA
Resting state	63.95 ± 1.29	72.32 ± 1.21	28.73 ± 2.61	40.61 ± 1.75	27.71 ± 2.72	38.73 ± 2.50	283.60 ± 53.06	756.93 ± 96.63	546.73 ± 143.64	781.77 ± 93.70	1.17 ± 0.26	3.06 ± 0.21
Stroop congruent	67.20 ± 1.43	72.43 ± 1.24	26.94 ± 3.00	40.20 ± 2.19	30.35 ± 3.18	42.98 ± 3.20	341.48 ± 63.75	697.93 ± 112.04			0.86 ± 0.21	2.60 ± 0.20
Stroop incongruent	68.14 ± 1.42	72.54 ± 1.23	23.94 ± 1.85	40.91 ± 1.95	25.95 ± 2.14	41.76 ± 2.63	218.08 ± 68.93	635.10 ± 83.54			0.70 ± 0.20	2.83 ± 0.22
Stroop mix	66.94 ± 1.32	73.02 ± 1.20	26.57 ± 2.91	42.98 ± 2.20	26.80 ± 2.79	42.46 ± 2.84	314.59 ± 70.93	666.32 ± 97.66			0.86 ± 0.23	2.63 ± 0.21
0-back	63.83 ± 1.23	71.12 ± 1.25	28.73 ± 3.76	42.12 ± 2.41	30.98 ± 3.81	43.94 ± 3.24	386.29 ± 89.86	752.55 ± 109.00			0.95 ± 0.21	2.39 ± 0.16
1-back	64.27 ± 1.28	72.16 ± 1.23	28.29 ± 3.31	42.29 ± 1.94	30.25 ± 3.38	42.25 ± 2.43	323.65 ± 63.42	712.30 ± 92.67			0.92 ± 0.20	2.45 ± 0.15
2-back	65.60 ± 1.21	72.08 ± 1.35	29.13 ± 3.20	39.89 ± 2.10	29.82 ± 3.52	40.74 ± 2.90	377.61 ± 103.86	622.37 ± 86.54			0.82 ± 0.18	2.27 ± 0.19
Recovery	63.13 ± 1.36	71.72 ± 1.27	26.09 ± 2.46	40.98 ± 1.94	25.78 ± 2.54	37.85 ± 2.00	257.04 ± 51.18	640.73 ± 76.16	448.84 ± 685.49	945.12 ± 126.95	0.83 ± 0.22	2.89 ± 0.24

HF: high frequency, mHR: mean heart rate, OA: older adults, RMSSD: root mean square of successive NN interval differences, YA: young adults.

**Table 3:** Interaction *p*-values (group as between-participants factor).

	Resting State					Recovery				
	mHR	SDNN	RMSSD	HF	D2	mHR	SDNN	RMSSD	HF	D2
Stroop congruent	0.004 **	0.530	0.567	0.122	0.568	0.004 **	0.556	0.871	0.786	0.260
Stroop incongruent	<0.001 ***	0.032 *	0.103	0.472	0.424	<0.001 ***	0.409	0.241	0.644	0.838
Stroop mix	0.024 *	0.098	0.097	0.211	0.638	0.005 **	0.621	0.181	0.715	0.303
0-back	0.239	0.686	0.552	0.386	0.149	0.114	0.520	0.773	0.840	0.040 *
1-back	0.621	0.455	0.756	0.467	0.259	0.365	0.734	0.978	0.951	0.079
2-back	0.093	0.667	0.974	0.030 *	0.157	0.021 *	0.141	0.719	0.141	0.042 *

HF: high frequency, mHR: mean heart rate, RMSSD: root mean square of successive NN interval differences, SDNN: standard deviation of the NN intervals. * *p* < 0.05; ** *p* < 0.01; *** *p* < 0.001.

## Data Availability

Data are available from the corresponding author upon reasonable request.
